# Explaining decisions of deep neural networks used for fish age prediction

**DOI:** 10.1371/journal.pone.0235013

**Published:** 2020-06-19

**Authors:** Alba Ordoñez, Line Eikvil, Arnt-Børre Salberg, Alf Harbitz, Sean Meling Murray, Michael C. Kampffmeyer

**Affiliations:** 1 Department of Statistical Analysis, Machine Learning and Image Analysis, Norwegian Computing Center, Oslo, Norway; 2 Department of Deep-Water Species and Cartilaginous Fish, Institute of Marine Research, Tromsø, Norway; 3 Department of Physics and Technology, University of Tromsø, Tromsø, Norway; Department of Agriculture, Water and the Environment, AUSTRALIA

## Abstract

Age-reading of fish otoliths (ear stones) is important for the sustainable management of fish resources. However, the procedure is challenging and requires experienced readers to carefully examine annual growth zones. In a recent study, convolutional neural networks (CNNs) have been demonstrated to perform reasonably well on automatically predicting fish age from otolith images. In the present study, we carefully investigate the prediction rule learned by such neural networks to provide insight into the features that identify certain fish age ranges. For this purpose, a recent technique for visualizing and analyzing the predictions of the neural networks was applied to different versions of the otolith images. The results indicate that supplementary knowledge about the internal structure improves the results for the youngest age groups, compared to using only the contour shape attribute of the otolith. However, the contour shape and size attributes are, in general, sufficient for older age groups. In addition, within specific age ranges we find that the network tends to focus on particular areas of the otoliths and that the most discriminating factors seem to be related to the central part and the outer edge of the otolith. Explaining age predictions from otolith images as done in this study will hopefully help build confidence in the potential of deep learning algorithms for automatic age prediction, as well as improve the quality of the age estimation.

## Introduction

**Fig 1:**
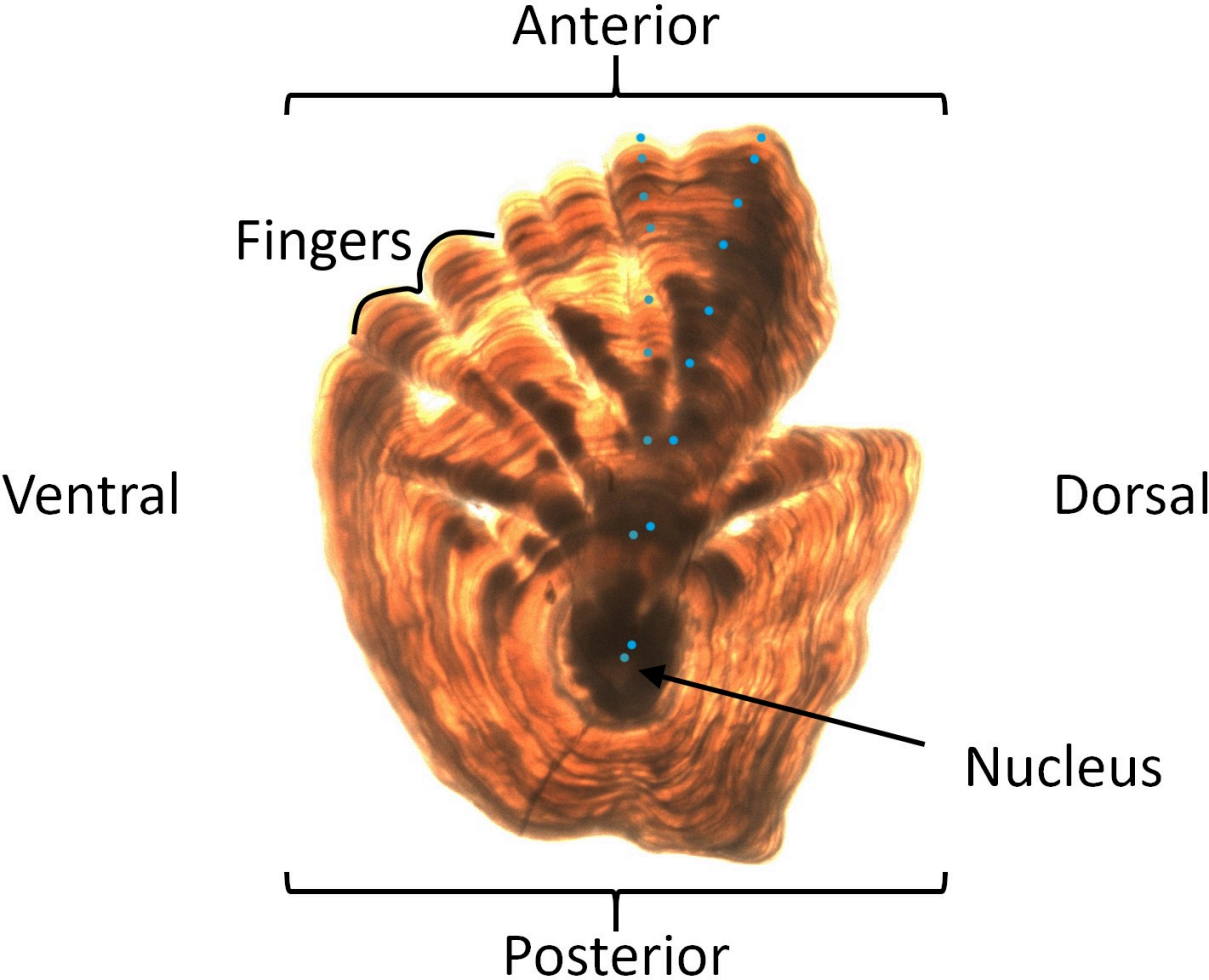
Right Greenland halibut otolith image. The main structural parts of the otolith are indicated together with annotated manual year zone readings in turquoise dots. Both alternatives predict an age of 8 years. Photo was provided by Kristin Windsland, Norwegian Institute of Marine Research.

The reliable estimation of the age distribution of fish stocks is an important aspect in marine research and resource management in order to maintain sustainable fisheries. A bottleneck in this area is the complicated task of age-reading of individuals. One of the procedures used for age-reading requires human experts to examine images of otoliths or ear stones (i.e. calcified structures located in the inner ear of bony fish). For many stocks, specialists are trained to carefully examine incremental daily and annual growth in the otoliths [1] and may sometimes use additional auxiliary data [2] (e.g. fish size, date of capture, sex, etc.). The annual growth zones can be particularly difficult to identify and separate in the otolith structure (Fig 1), and this examination process has proved to be expensive and time-consuming. Nonetheless, on the order of a million otoliths from captured fish are read annually on a global basis [3]. Consequently, methods for automating age-reading from otolith image samples have been proposed. For example, Moen et al. [4] recently estimated the age of Greenland halibut (*Reinhardtius hippoglossoides*) by utilizing a convolution neural network (CNN). The presented results were promising since the deviation between the age predictions by the CNN and the ages read by experienced readers was comparable to reported differences between human age-reading experts. However, for the age-reader community and fisheries managers, confidence in these deep learning algorithms through some level of decision understanding is still needed. As highlighted in Lapuschkin et al. [5], it seems especially important to verify that the deep learning models do not learn biased prediction rules based on noise or other irrelevant imaging artefacts present in the data.

When it comes to images, the explanation of decisions from deep learning algorithms is often a matter of visualization. Visualizing what a neural network has learned is an active field of research, and methods can be broadly separated into two categories: (i) perturbation-based methods and (ii) back-propagation-based methods. In all of these methods, explanations of neural network decisions are represented as visual heatmaps. The fundamental idea behind perturbation-based visualization methods (e.g. [6,7]) assumes that making some alterations to the pixels contributing the most to the predictions (e.g. by using occlusion patches), will cause a significant drop in the probability of the predicted class. The major problem with these methods is that they are computationally expensive. Visualizing what the neural network has learned for a single image requires running multiple forward passes of the model. This may not be feasible if thousands of images need to be analyzed. In this case, back-propagation-based visualization methods may be better suited. Some of these approaches measure the change of the output with respect to variations in the input space, as measured by the gradient (e.g. [6,8,9,10]) and the backpropagation of this quantity through non-linear layers varies between the different techniques. Layer-wise relevance propagation (LRP) as presented in Bach et al. [11] is yet another back-propagation based visualization method. It aims to assign the importance of an input pixel to the overall output prediction score by back-propagating a relevance score encoding the information about the model’s decision.

An important objective of this work is to identify the image features CNNs use to predict different ages or age intervals. Because the resolution of the images is substantially reduced to efficiently train CNNs, the annual zones are blurred. Thus, it is natural to expect that features other than the annual zones read by human readers will be utilized. We want to know to what extent attributes such as size, contour shape (henceforth denoted as *shape*) and inner structure of the otolith are meaningful for the neural network. Therefore, we train models using different versions of the otolith images and compare performance. Further, we visualize and analyze neural network predictions for otoliths with different ages. We have chosen the state-of-the-art LRP technique since it allows for an efficient and better identification of relevant pixels [12] and is less exposed to noise [13], therefore producing more consistent visual heatmaps than other methods. In order to practically characterize relevant features of all the otoliths within a specific age range, (i) we apply clustering of the LRP visual explanations by adopting a pipeline presented in Lapuschkin et al. [5] and (ii) we compute some average heatmaps. This allows us to identify patterns observed in the growth of Greenland halibut, and to correlate specific age ranges with areas of the otoliths where the network focuses without having to screen through all the individual heatmaps. With these experiments, we are able to investigate the attributes used by neural networks and to identify the particular areas of the otoliths used to differentiate certain age ranges.

## Materials and methods

### Data and modeling choices

The available otolith images were provided by the Norwegian Institute of Marine Research that launched a data collection program on Greenland halibut between 2006 and 2017. The same data were used by Moen et al. [4] in their automatic age determination of Greenland halibut otoliths using deep neural networks, and no permits were required to further study the published data. Two different experts from the Norwegian Institute of Marine Research conduced the age-readings, but each otolith sample was only read by one of the two experts. The fish ages varied between 1 and 26 years (as determined by the readers).

**Fig 2:**
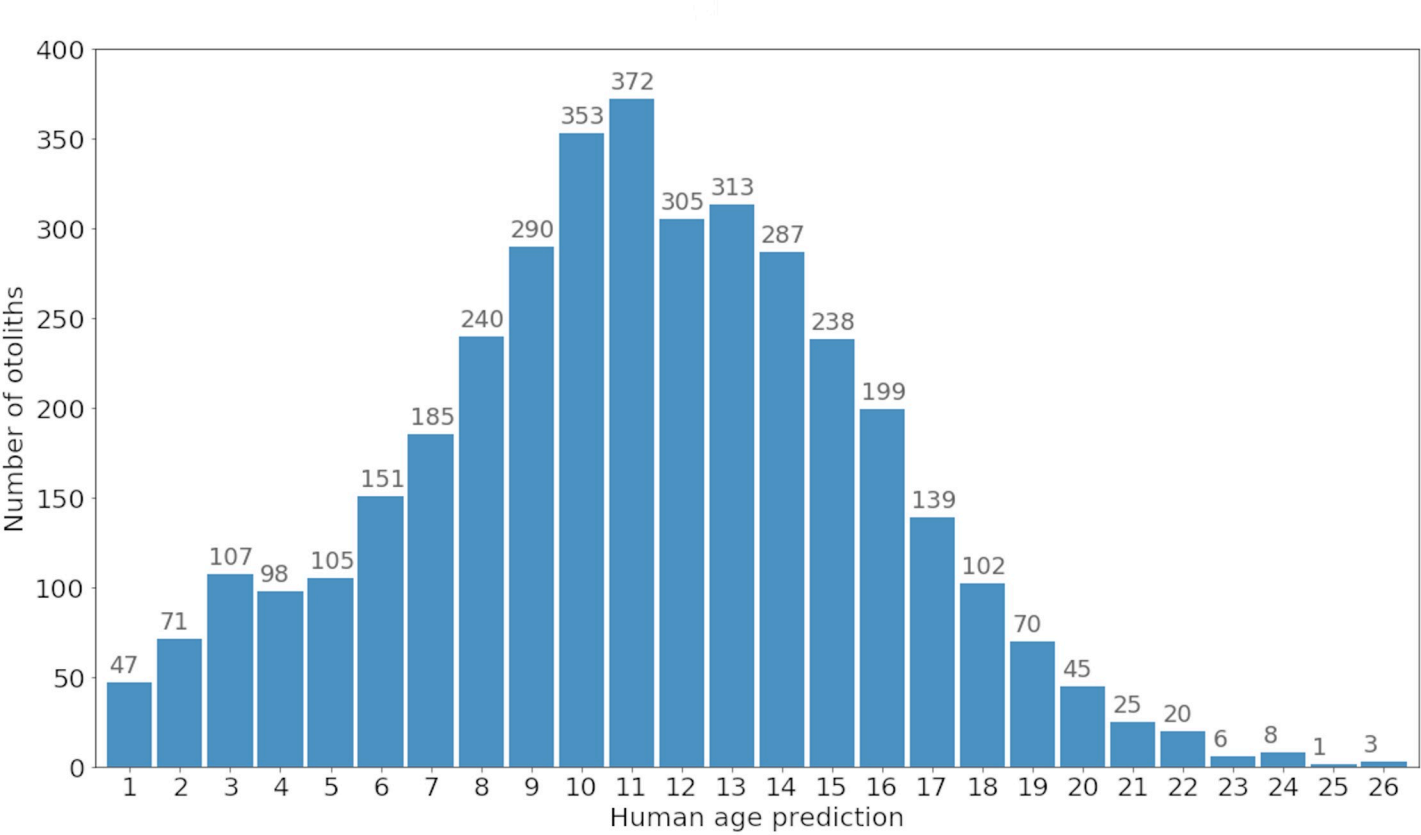
Age frequency distributions predicted by human experts in the training set.

The images originally were composed of paired otoliths and after processing them to separate the right one from the left, a regression model based on the Inception v3 architecture [14] was trained in Moen et al. [4]. We decided, however, to regard the age determination problem as a classification problem in this study. We found that the task of explaining deep learning predictions was well explored for classification networks, but not for regression networks. As the age regression problem can easily be redefined to account for age classes, we therefore trained a model to classify ages into 26 categories (from 1 to 26 years). We identified the VGG19 network [15] to be well suited for this task since we obtained very similar results to that of the original regression network [4]. We experimented with 3945 image samples of the right otolith database of which 3780 samples were the basis of our training dataset and 165 samples constituted the test set. Note that the manually estimated age distribution for our training images (Fig 2) was very comparable to the corresponding age distribution presented in Moen et al. [4].

The original image samples of the right otolith had a resolution of 2596x1944 pixels which we resized to fit a square with the default input size for the VGG-19 model (224x224). Note that in Moen et al. [4] the images were also resized, choosing a 400x400 square. In both of these image rescaling processes, details of the annual growth zones visible in the original images were lost. However, training a network without resizing the images would have demanded more memory than available for common graphics processing units, commonly needed for training deep neural networks.

**Fig 3:**
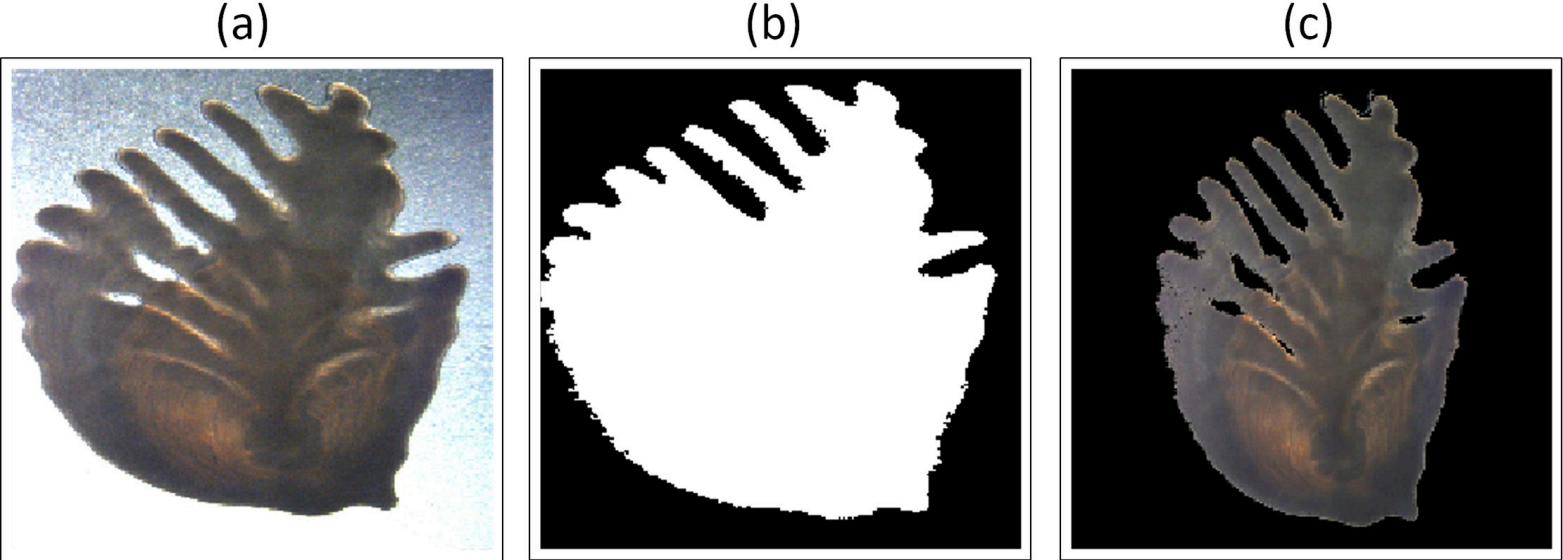
Different versions of otolith images for a 13 years old fish. (a) Baseline image, (b) Binary image, (c) Standardized image.

We were particularly interested in investigating the relative importance of the size, shape and inner structure attributes of the otolith. For this purpose, we trained models using three different versions of the otolith images that we denoted *baseline*, *binary* and *standardized data* (Fig 3). The baseline data (Fig 3A) corresponded to the original right otolith images, resized to 224x224. The binary data (Fig 3B) were created by modifying the baseline data through setting all otolith pixels to one and all background pixels to zero. This allowed us to investigate the importance of the inner structure of the otoliths and see how the network would respond when only using size and shape attributes. Finally, for standardized data (Fig 3C) the background pixels were set to zero and the size of the otoliths (in the vertical axis) was standardized. This enabled us to investigate the importance of removing the size attribute, while keeping information on the shape and inner structure.

For the three considered data types, the VGG-19 models were trained using the pre-trained weights from the image database ImageNet [16] as initialization. Like in Moen et al. [4], we did some data augmentation by randomly rotating the input images between 0 and 360 degrees, applying random horizontal and vertical flips and randomly shifting the images by +/- 10 pixels in the vertical direction. We also normalized the images by subtracting a channel-wise mean derived from the training images and dividing by the standard deviation. This was done for both the test and the training set. For the training process, we used the following set of hyperparameters: the Adam optimizer function, a batch size of 8, a learning rate of 0.0004 and no weight decay. Note that we optimized over the cross-entropy loss during training.

### Grouping ages

We classified images into one of 26 age classes and, to draw more general conclusions across ages, we found it useful to do further analysis on coarser age groups. The Greenland halibut otolith grows asymmetrically from the nucleus with age, with modest growth in the posterior direction and significant growth in the anterior direction with distinguishable fingers that grow longer and more distinct with age [17]. We decided to exploit these characteristics for grouping the ages.

**Fig 4:**
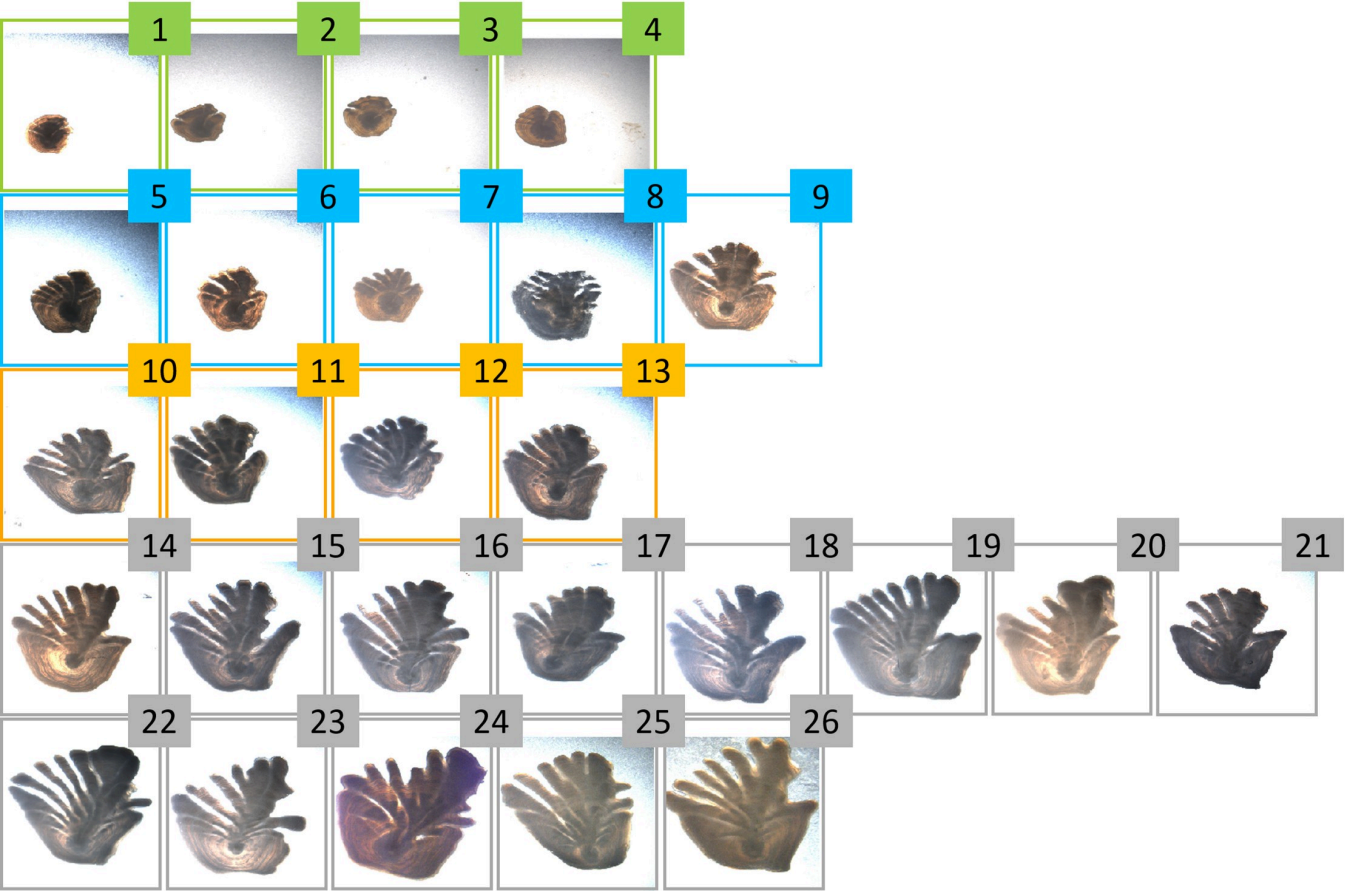
Examples of baseline otolith images from the training set for the different ages predicted by human readers. Otoliths belonging to juveniles, adolescents, young adults and adults are surrounded by green, blue, orange and grey rectangles, respectively.

Fig 4 presents one otolith sample from our baseline database for each age predicted by a human expert (from 1 to 26). Following some terminology used in Albert [18], we subdivided the different age categories into four groups:

- Juveniles (ages: 1–4): the otoliths do not have clearly separable fingers, have a smooth and nearly circular shape and a large nucleus relative to the size of the otolith.
- Adolescents (ages: 5–9): the otoliths have clearly distinguishable fingers, but they are relatively short, with a considerably greater growth in the anterior direction above the nucleus compared to the posterior growth below the nucleus.
- Young adults (ages: 10–13): the otoliths have longer fingers than the previous category.
- Adults (ages: 14–26): the otoliths are characterized by fingers that have grown significantly and their shapes have been altered by the asymmetric growth.

### Analysis of model performance

We compared our results with the results of Moen et al. [4] by applying the root mean squared error (*RMSE*) between age prediction and read age, since this has a more intuitive and useful unit of age than the mean squared error used in the original paper. Like Moen et al. [4], we also considered the coefficient of variation (*CV*) of independent estimators (human and CNN) computed from each individual otolith. This metric was averaged across the otoliths for the entire dataset to obtain the mean coefficient of variation ($\bar{CV}$). The latter is typically used as a between-reader uncertainty measure when evaluating human versus human precision for age determination (e.g. [19,20]). Note that between-reader biases may substantially increase the $\bar{CV}$.

For the age classification network, we analyzed the relative importance of shape, size and inner structure for the different age groups (juveniles, adolescents, young adults and adults) by looking at the model performance expressed in terms of $\bar{CV}$. We performed our experiments on the training as well as the test sets, since the sample size of the latter (165) was too small to generalize our results. When examining individual *CV* values across age groups, we observed some outliers that could slightly skew the $\bar{CV}$. We therefore applied the interquartile range rule [21] (also known as 1.5xIQR rule) to exclude the outliers from the *CV* dataset and then computed the associated $\bar{CV}$ for each age group.

### Visualizing explanations with LRP

In order to visualize neural network predictions on the selected right otolith image database, we used the implementation of the LRP developed by Alber et al. [22] and available at: https://github.com/sebastian-lapuschkin/lrp_toolbox. The implementation is based on the deep learning library Keras [23].

**Fig 5:**
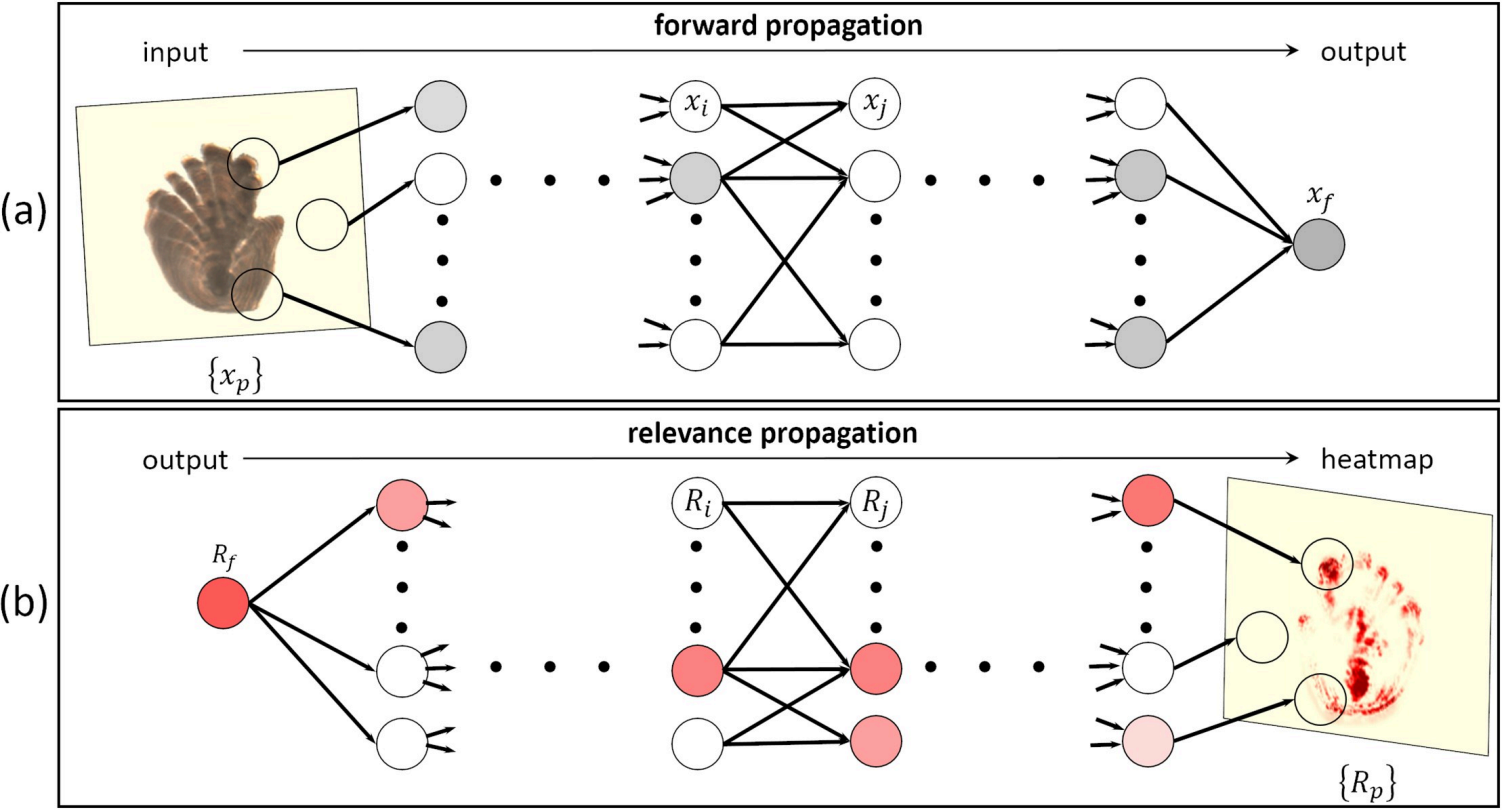
Illustration of the LRP conceptual flow applied to an otolith image {*x*_*p*_} from a 13-year-old fish (inspired from Montavon et al. [24]). In the forward propagation phase (a), the output neuron of the network *x*_*f*_ has retained the evidence of the actual age class. In the relevance propagation phase (b), this output is first attributed the relevance score *R*_*f*_ before being redistributed backward in the network. The relevance scores of all the pixels can be visualized as a heatmap {*R*_*p*_} that can have different characteristics depending on the chosen propagation rule. Here, the relevant pixels are highlighted in red and contribute positively to the prediction. The higher the degree of red, the more positive the contribution of the pixel to the prediction.

The LRP visualization technique is illustrated in Fig 5, using an example of an otolith image. A function *f* is trained to map an age class to the image, where the image is decomposed into a set of pixel values ***x*** = {*x*_*p*_}, the index *p* denoting a specific pixel (Fig 5A). After forward-propagation, the output neuron of the network *x*_*f*_ has retained the evidence of the actual age class and contains the encoding from the function *f*. LRP aims to associate each pixel *p* with a relevance score *R*_*p*_ illustrating the contribution of a pixel to the decision of the neural network. To obtain this, the relevance score *R*_*f*_ is first attributed to the output neuron *x*_*f*_, so that *R*_*f*_ = *x*_*f*_. The LRP algorithm then redistributes the relevance *R*_*f*_ backward to all the neurons until reaching the input (Fig 5B). Note that the redistribution process should satisfy a relevance conservation process so that the sum of relevance values per layer are preserved. At the input level, the relevance scores of all the pixels are represented by a visual heatmap {*R*_*p*_}, also called a relevance map. In Fig 5, the first neuron of the first hidden layer is perceived as strongly relevant by higher layers and therefore gives an important relevance to the pixels it is related to. The information exchanged by neuron *i* and *j* in two succeeding layers is referred to the message *R*_*i*←*j*_ and is generally written as (e.g. [5]):

$$R_{i\leftarrow j}=\frac{q_{ij}}{\sum_{i}q_{ij}}R_{j},$$ (1)

where *q*_*ij*_ denotes the contribution of neuron *i* for activating neuron *j* and *R*_*j*_ denotes the neuron *j* relevance score. Then, the score *R*_*i*_ associated with neuron *i* is retrieved by summing all messages coming from the higher-layer neurons to which neuron *i* made a contribution. This can be expressed as:

$$R_{i}=\sum_{j}R_{i\leftarrow j}=\sum_{j}\frac{q_{ij}}{\sum_{i}q_{ij}}R_{j}.$$ (2)

The contribution *q*_*ij*_ of neuron *i* for activating neuron *j* can be calculated by using different propagation rules, the most commonly used being reported in Montavon et al. [25]. Let the neuron activation *a*_*j*_ of a neural network be expressed by the following equation:

$$a_{j}=\sigma \left(\sum_{i}a_{i}w_{ij}+b_{j}\right),$$ (3)

where *σ* is a monotonically increasing nonlinear function, *a*_*i*_ is the neuron input and *w*_*ij*_ and *b*_*j*_ are the learned weights and bias parameters, respectively. One of the rules satisfying local conservation properties and working well in practice is the *αβ*-rule (e.g. [13]):

$$R_{i}=\sum_{j}\left(\alpha \frac{a_{i}w_{ij}^{+}}{\sum_{i}a_{i}w_{ij}^{+}}-\beta \frac{a_{i}w_{ij}^{-}}{\sum_{i}a_{i}w_{ij}^{-}}\right)R_{j},$$ (4)

where the superscripts ^+^ and ^−^ express the positive and negative parts, respectively. The parameters *α* and *β* are scalars and are chosen such that *α*−*β* = 1 and *β*≥0. Note that this relevance decomposition is a combination of positive (activating) and negative (inhibiting) contributions. It is possible to ignore the inhibiting contributions by setting *β* = 0. This corresponds to the denoted LRP-*α*_1_*β*_0_ rule and can be simply written as:

$$R_{i}=\sum_{j}\frac{a_{i}w_{ij}^{+}}{\sum_{i}a_{i}w_{ij}^{+}}R_{j}.$$ (5)

We used the LRP- *α*_1_*β*_0_ rule in our experiments to only focus on activating contributions of the CNNs.

### Analyzing visualization results

To establish a possible correlation between specific age groups and otoliths areas where the network focused, we could have chosen to screen through all the individual relevance maps derived from the aforementioned LRP-*α*_1_*β*_0_ rule. However, this process would have been very time consuming and meaningful conclusions across classes and age groups would have been difficult to draw. Instead, we carried out a two-step analysis. First, we applied clustering on the relevance maps and visualized the results by performing dimensionality reduction. Then, we computed some average relevance maps to identify pixel activation patterns that were characteristic of an age group. To facilitate the analysis, we only considered two consecutive age groups at a time. We therefore performed three distinct experiments, one for each pair of consecutive age groups, i.e.: juveniles + adolescents, adolescents + young adults and young adults + adults. We decided to concentrate on data where the internal structure was available (i.e. baseline and standardized data) and did not consider binary data. The reason for this was that the relevance maps associated with binary data varied by intensity just along the contour of the otolith, thereby only providing information about the shape.

With the first step of the analysis, we aimed to evaluate whether the relevance maps from otolith images belonging to the same age group clustered together. Different approaches could have been used for this, for instance a combination of standard K-means clustering [26] and principal component analysis [27]. For analyzing relevance maps, Lapuschkin et al. [5] presented what they called a spectral relevance analysis (SpRAy) pipeline. We used the same approach as them that consisted of applying spectral clustering followed by t-distributed stochastic neighborhood embedding (t-SNE) [28] for dimensionality reduction and visualization. We decided to choose this method given that spectral clustering presents some interesting analytic properties. For example, there is no assumption on the shape and size of the clusters contrary to K-means. Moreover, t-SNE tends to provide better visualizations than principal component analysis, especially when dealing with non-linear manifold structures. The SpRAy pipeline that we used consisted of the following steps:

- Pre-processing: We did an initial cropping tightly along the otolith contour of the relevance maps resulting from LRP, to eliminate contributions from background noise. Afterwards, we resized all the relevance maps to the same size and also did a final downsampling to obtain heatmaps of 56x56 by summing pixels over a regular grid. The downsampling helped to accelerate the clustering and led to more robust results, as also pointed out in Lapuschkin et al. [5].
- Spectral Clustering: The 56x56 relevance maps from the pre-processing step were flattened to one-column vectors and stacked horizontally to form a matrix. The latter was used to compute an affinity matrix using a *k*-nearest neighbor graph, following the recommendations from Von Luxburg [29]. The parameter *k* for the *k*-nearest neighbor was chosen to be equal to log(*n*), where *n* was the considered number of samples. Then, spectral clustering was applied to a projection of the normalized Laplacian computed from the affinity matrix. In our experiments, we investigated clustering results for pairs of consecutive age groups. Thus, the number of clusters was set to 2 for each experiment.
- Visualization of results: Finally, we visualized the results with t-SNE choosing two dimensions for the embedded space. Embedding coordinates were computed on pair-wise distances derived from the affinity matrix used for the clustering.

We computed the clustering accuracies resulting from the above process by comparing the cluster label assignments from SpRAy with the model predictions assigned to age groups. This was measured in terms of F1-scores [30], where a high F1-score referred to a high clustering accuracy and thereby a good separation between age groups based on distinguishable pixel activation patterns.

Regarding the second step of the analysis, we computed an average relevance map for each predicted age, using the 56x56 heatmaps derived from the pre-processing step of SpRAy. In this way, we could check consistencies with the patterns observed in the growth of Greenland halibut otoliths and we could identify whether otoliths of different ages shared the same type of characteristics in terms of activated pixels. Note that the otolith samples were oriented in the same way, i.e. with the anterior part pointing upwards (Fig 4). Thus, to extract the general pixel activation pattern, it was not considered necessary to account for variations in the positioning of the otolith in the image.

## Results and discussion

**Fig 6:**
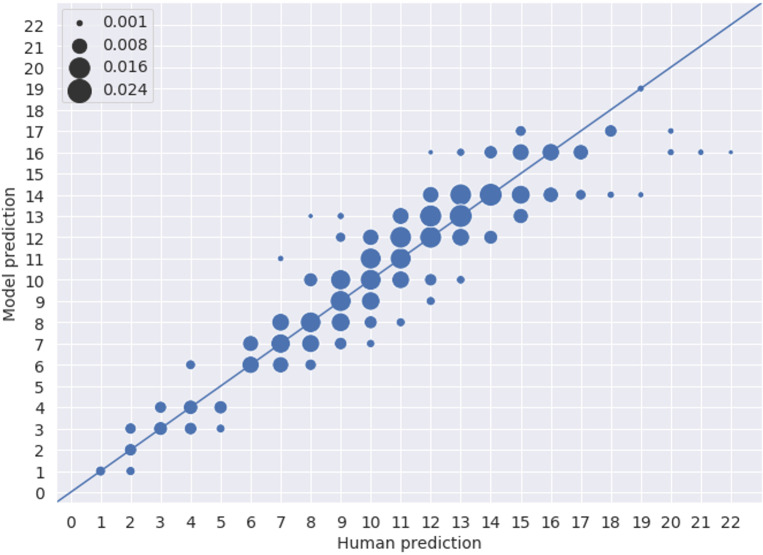
Age predictions of model vs human expert for the test set based on classification using baseline data. The scatters have a radius proportional to the probability density of data. This result can be compared with Fig 5 in Moen et al. [4], where the authors observed an underestimation of ages predicted by the model relative to human readers for the right otoliths.

The results achieved by applying the trained VGG19 model to classify the baseline test set into 26 categories (from 1 to 26 years) were very similar to the results obtained by Moen et al. [4] (Table 1). We also noticed the same pattern of underestimation for the older age groups compared to the human readers (Fig 6). These findings verified that age prediction may also be done in a classification setting.

**Table 1:** Comparison of performance achieved on the test set composed of right otoliths in Moen et al. [4] and our classification results obtained on the baseline test set.

Model	Inception v3 Moen et al. [4]	VGG19 Baseline
*RMSE*	1.65	1.69
$\bar{CV}$ (%)	8.97	9.0

**Fig 7:**
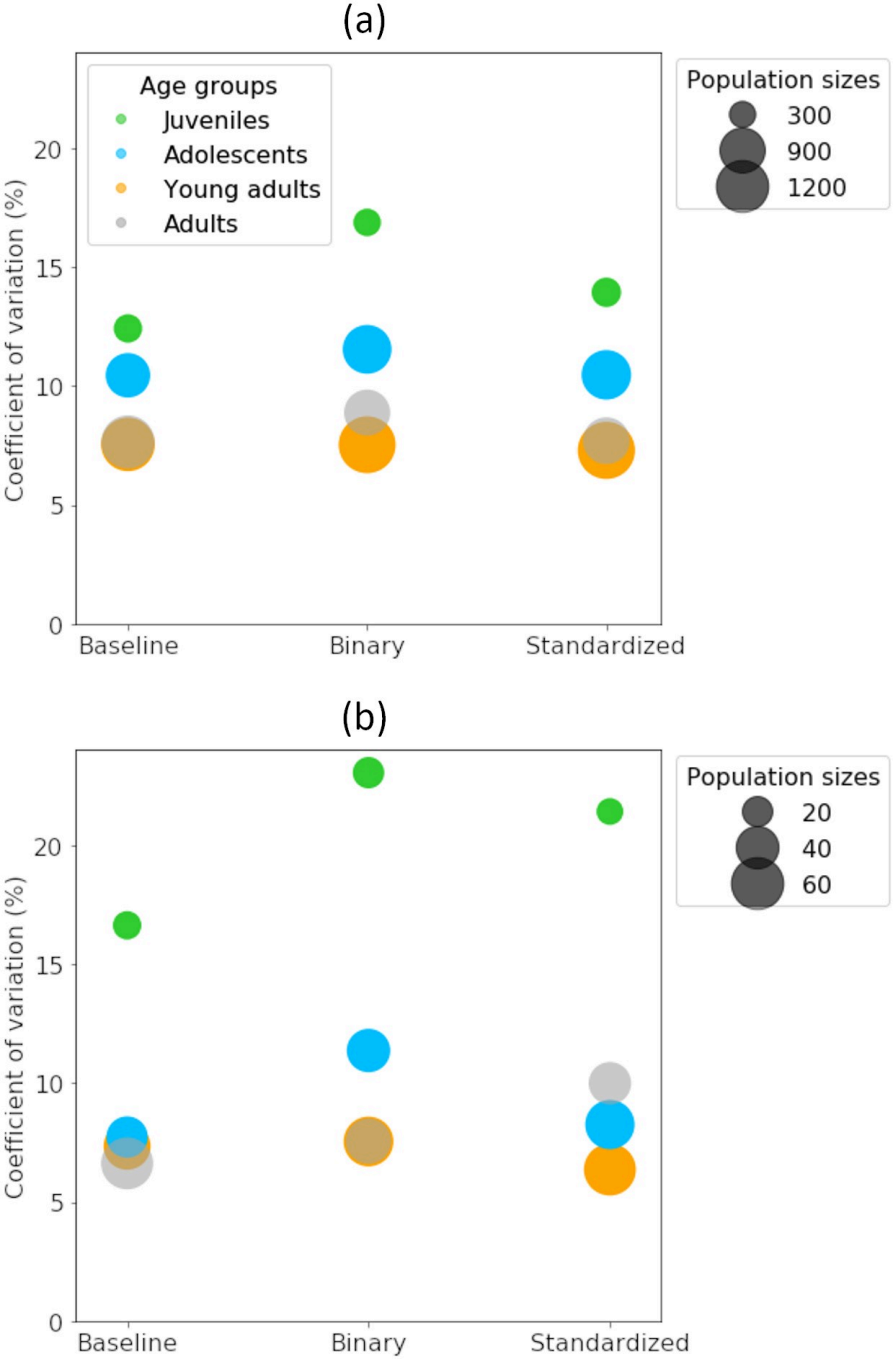
$\bar{CV}$ computed from the training (a) and test (b) datasets across the different age groups (juveniles, adolescents, young adults and adults) and for the different versions of the data (baseline, binary and standardized). The different age groups are associated with different colors and the value of $\bar{CV}$ is represented by a circle having a radius proportional to the predicted population size.

The general performance of the model evaluated in terms of $\bar{CV}$ varied among age groups and data types (Fig 7A and 7B). For juveniles, the best performance was achieved when both information about inner structure and size were included (baseline dataset), while the removal of inner structure (binary) had the largest negative impact on the $\bar{CV}$. This trend was also similar for adolescents and adults, although to a lesser extent. For young adults, the removal of inner structure and size did not seem to affect the $\bar{CV}$ at all. Hence, shape seemed most important for age estimation within this group.

The degree of separation between pairs of consecutive age groups based on relevance maps and F1-scores varied across the considered age groups (Table 2). The pair juveniles + adolescents had the largest F1-scores, therefore showing a good separation of age groups for both baseline and standardized data. This also implied the existence of distinct pixel activation patterns to separate these age groups based on the relevance maps. The lower F1-scores obtained for the other pair-comparisons indicated that it was harder to separate older fish age groups. In the three pair-comparisons, the standardized F1-scores were higher than the baseline F1-scores, suggesting that the CNN picked up more distinct activation patterns with age when using standardized data. More detailed illustrations of the clustering results through t-SNE visualizations can be found in S1–S6 Figs.

**Table 2:** Summary of the clustering accuracy scores for the different age groups considering baseline and standardized data (including training + testing).

Age groups	Juv.–Ado.	Ado.–Y-adult	Y-adult–Adult
F1-score *Baseline*	0.88	0.52	0.66
F1-score *Standardized*	0.91	0.59	0.74

**Fig 8:**
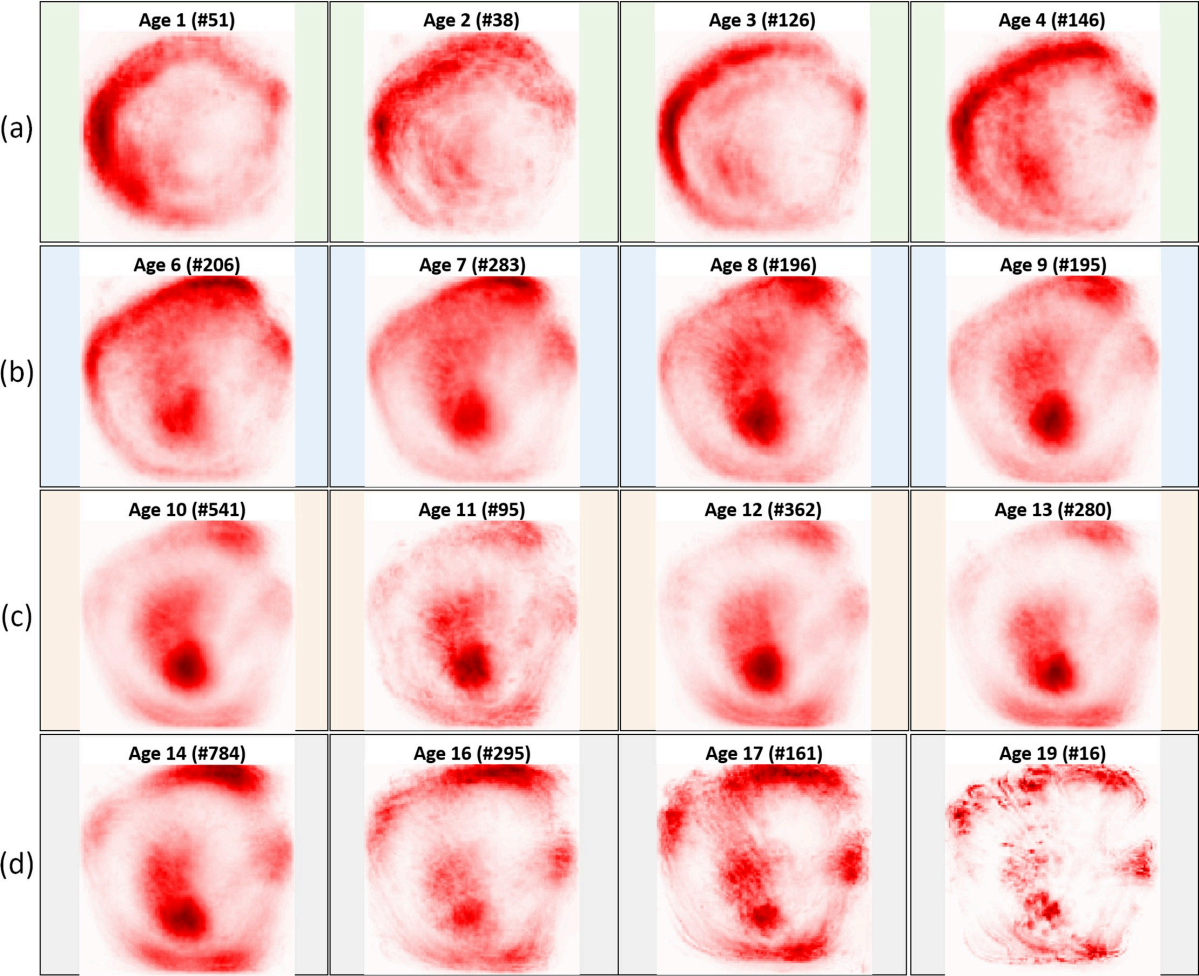
Average relevance maps computed from the baseline data considering different predicted ages belonging to (a) juveniles, (b) adolescents, (c) young adults and (d) adults. The number of samples belonging to a given predicted age are also indicated in the upper part of the average image. For each age group, only the four ages having the higher number of predictions had their average relevance map displayed. Note that each heatmap has been normalized by its maximum and the higher the degree of red, the more positive the contribution of the pixel to the prediction.

**Fig 9:**
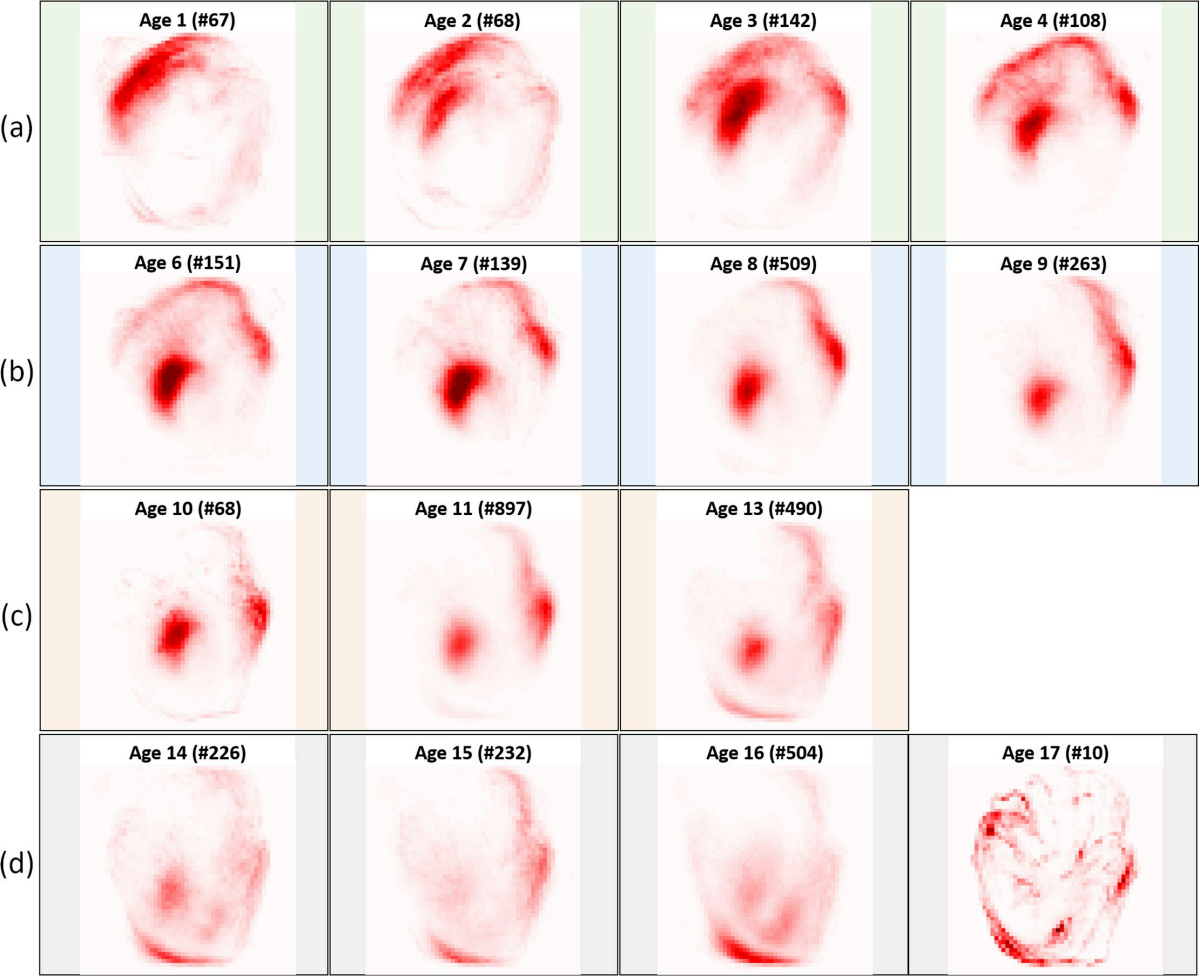
Average relevance maps computed from the standardized data considering different predicted ages belonging to (a) juveniles, (b) adolescents, (c) young adults and (d) adults. The number of samples belonging to a given predicted age are also indicated in the upper part of the average image. For each age group, only the four ages having the higher number of predictions had their average relevance map displayed, except for the young adults group where only ages 10, 11 and 13 were predicted. Note that each heatmap has been normalized by its maximum and the higher the degree of red, the more positive the contribution of the pixel to the prediction.

The average relevance maps (Figs 8 and 9) were consistent with the general pattern change observed in the growth of Greenland halibut otoliths (Fig 4), i.e. the otolith started with a symmetric, nearly circular shape and grew asymmetrically with age. The greatest growth was in the anterior direction and the distinct activation in the nucleus became smaller relative to the size of the otolith. In both the baseline and standardized data, the most important factors for differentiating between pairs of consecutive age groups were related to the nucleus and the outer edge activations of the otolith. For the juveniles in the baseline data (Fig 8A), there was an activation on the left corner of the nucleus, while for the adolescents (Fig 8B), there was more localized activation in the anterior and the center of the nucleus. The corresponding results for the standardized data (Fig 9) showed a similar pattern; the juveniles presented an activation on the upper left corner of the nucleus (Fig 9A) and the network concentrated more on the ventral contour, while the adolescents (Fig 9B) had a stronger focus on the anterior/dorsal and on the left part of the nucleus. For adolescent + young adults and young adults + adults, it was harder to identify differentiating otolith areas to separate age groups based on the average relevance maps (Figs 8B, 8C, 8D, 9B, 9C and 9D). These older age groups in the baseline data presented activation in the interior part of the otolith between the nucleus and the anterior direction. This was not the case in the standardized data. In addition, we saw a small difference between the young adults (Fig 8C) and the adolescents (Fig 8B) in the baseline data. The young adults had a slightly more localized activation in the anterior and the nucleus, compared to the adolescents. The activations around the upper part of the fingers and the posterior were most evident for the adults (Fig 8D), while the network seemed to focus more on the nucleus for the young adults (Fig 8C). For the standardized data, the dorsal part of the otolith seemed to get slightly more localized activations for the young adults (Fig 9C) compared to the adolescents (Fig 9B). For the adults, the activations on the posterior parts of the otoliths were more accentuated (Fig 9D), while for the young adults the dorsal part and the nucleus were the main focus of the network (Fig 9C).

The differences in activations detected in the relevance maps (Table 2, Figs 8 and 9) were most pronounced between the juveniles and the adolescents, where the most differentiating component appeared to be the distinct parts of the nucleus. The juveniles have a large nucleus relative to the size of the otolith (Fig 4) and our results showed that removing information about the internal structure (i.e. using binary data) led to poor model performance (Fig 7). This suggested that the nucleus was a key component of the otolith for the youngest age group. For older age groups, the network focused on various parts of the otolith contour (Figs 8 and 9) further supporting that the shape was an important attribute for achieving good separation within these age groups (Fig 7).

Furthermore, we noticed that for standardized data, the ventral and dorsal parts were more enhanced depending on the age group and the left part of the nucleus was given a more important focus (Fig 9), compared to the baseline data (Fig 8). One limiting factor of heatmaps is that there is no ground truth. Consequently, we do not know whether the pixel activation patterns extracted from the baseline data were more relevant than the ones from the standardized data. The robustness of the activation patterns may be assessed by applying a region perturbation strategy, as introduced in Samek et al. [12] consisting of modifying the otolith areas that were considered as important by the neural network for the different data types. Additional representations of the otolith images (e.g. [31]) may also be included in this type of analysis.

In the present study, we were particularly interested in verifying that the trained classifier picked up relevant otolith image features and did not learn a biased prediction rule based on noise or some artefact related to the acquisition of the image (e.g. [5]). Therefore, we did not focus on improving the quality of the trained neural network. However, the computation of heatmaps depends on the quality of the model. The better the model is at predicting fish age, the more likely the pixel activation patterns will be meaningful and support the model decision. This in turn will result in more realistic (and probably better) differences across ages identified by the heatmaps. Although we had an adequate amount of training samples in the adult age group (Fig 2), we observed an underestimation of age predictions (Fig 6) and activations of the posterior pixels for both baseline and standardized data (Figs 8D and 9D). This is in contrast to the findings of Albert et al. [20] who noticed that the growth of the otolith was dominant in the anterior direction with hardly any growth in the posterior part of the right otoliths for older ages. Thus, it may be useful to investigate how the heatmap would change with better performing network architectures.

Along the same lines, pixel activation patterns can be biased if the manual readings used to train the CNN models are biased. A major drawback of the dataset used in this work is the lack of true ages for training the models. Albert [18] demonstrated that it was possible to access information about true time differences from a limited number of chemically marked juvenile Greenland halibut individuals that were tagged and injected with oxytetracycline some years before recapture. From the images of these otoliths, one could detect the location of the otolith contour at the time of injection and then determine exactly the number of annual zones to look for between the injection and time of recapture. More trustworthy CNN predictions and associated pixel activation patterns could be obtained by using this type of data for training the models.

Demystifying the prediction rule learned by neural networks is essential for building confidence in deep learning models. While manual age-readings from otoliths generally focus on annual growth zones, our results showed that neural network models provided reasonably good $\bar{CV}$ results on the baseline and standardized data (Figs 6 and 7), without enhanced activation of annual zone pixels (Figs 8 and 9). This lack of annual zone activation was expected due to the blurring caused by the reduction in resolution of the images used for efficient training of the CNN models. However, the fact that the CNNs picked relevant otolith image features, independent of what humans generally examine, indicated the potential to further use CNN age predictions. One approach could be to consider implementing a simple automatic system for imaging of otoliths followed by CNN age estimation, immediately after sampling otoliths in a lab. In this way, a coarse age distribution could be provided and used in e.g. stock assessment work, without waiting for the more tedious manual age readings.

In addition, simple age predictors like fish length or otolith pixel area, could be combined with the CNN results to improve the precision of the age distribution prediction. A weighted average of the predictors may possibly be more precise than each predictor. Note, however, that in order to combine different predictors to improve the precision, future work needs to verify that the considered predictors are independent and unbiased when being applied to the same samples. The independence between two different predictors can be examined by first computing the residuals (or difference) between the predictor age and the manual read age for each predictor and then looking at the correlation between the residuals of the two predictors. If the correlation coefficient is close to zero, the considered predictors can be considered independent. Further, a predictor is likely to be unbiased when the average residual value is close to zero. In our case, it is reasonable to believe that predictors based on biological size measures (such as fish length and otolith pixel area) are strongly correlated. Moreover, the CNN predictors based on the standardized images should be independent of size predictors, because size is eliminated through the standardization process. If the CNN predictor and a predictor based on size are unbiased and have comparable precision (e.g. measured in terms of *CV*), the variance of the average of these two predictors will decrease the variance to about half of the variance associated with each predictor. This will lead to a more precise age distribution.

The presented method, based on LRP and the subsequent SpRAy pipeline, can easily be adapted to other marine resource management tasks that could benefit from using deep learning. We can also use the method to understand the decision making of a CNN trained to predict the age of fish species other than Greenland halibut using otolith images but also e.g. fish scales of salmons that are captured under surveillance programs [32] or seal teeth images for which a larger amount of true age data are available [33]. The method could also be applied to the separation of different stocks [34] where it could be interesting to investigate the activation intensity along otolith contours based on binarized images. Distinct patterns would probably be observed for two different stocks.

Recall that the CNN activation patterns identified the nucleus and the outer edge of the otoliths as the most decisive factors for differentiating between pairs of consecutive age groups (Figs 8 and 9). These highlighted activation patterns could inspire the age-reading community to explore additional features to improve the manual readings.

Finally, even though the chosen LRP visualization approach seems promising for analyzing neural network decisions for otolith age prediction, it is worth mentioning that the implementation used in this study and developed by Alber et al. [22] comes with a toolbox that allows comparing different visualization methods. The toolbox includes the possibility of testing different LRP propagation rules [25] in addition to other back-propagation methods (e.g. [6,9]). A future task is to compare various methods and highlight whether they capture different otolith pixel activation patterns across age groups that could further help improve the manual analysis.

## Conclusion

This work explored the decision making of a neural network model trained to predict fish age from images of Greenland halibut otoliths. First, we showed that using a CNN classification network gave similar results to an earlier study (Moen et al. [4]) that used regression. Then, we used classification networks to investigate the relative importance of attributes such as shape, inner structure and size of the otolith by comparing the model performance of the original otolith images (baseline data) with that of images with no structure (binary data) and with no relative size information (standardized data). Our findings suggested that knowledge about the internal structure was the most important for the youngest age groups. Shape and size were, in general, sufficient for the older age groups. Finally, further analysis based on LRP and subsequent clustering showed that it was harder to separate the older fish age groups based on the relevance maps. Moreover, the strength and characteristics of the pixel activations varied between age groups, but the most discriminating factors seemed to be related to the nucleus and the outer edge of the otoliths.

We verified that the trained deep neural networks based their decisions on actual otolith characteristics and not on artefact related to the image acquisition process. The insights from this study pointed out some interesting features used by deep neural networks which may be used to further improve the quality of the age estimation. However, more importantly, the hope is that this study helps build confidence in deep learning methods for the purpose of age estimation from otoliths, thereby increasing the willingness to exploit such techniques to automate the process.

## Supporting information

S1 Fig t-SNE results for the pair juveniles + adolescents (baseline data). Cluster label assignments for classes: juveniles + adolescents using the baseline data. Center: Visualization by t-SNE. Each data point (i.e. colored circle) corresponds to a relevance map for one otolith and is overlaid with the predicted age. Darker colors on predicted ages refer to test samples. Outer images: Examples of otolith images overlaid by relevance maps for different ages predicted by the neural network.
(TIF)

S2 Fig t-SNE results for the pair juveniles + adolescents (standardized data). Cluster label assignments for classes: juveniles + adolescents using the standardized data. Center: Visualization by t-SNE. Each data point (i.e. colored circle) corresponds to a relevance map for one otolith and is overlaid with the predicted age. Darker colors on predicted ages refer to test samples. Outer images: Examples of otolith images overlaid by relevance maps for different ages predicted by the neural network.
(TIF)

S3 Fig t-SNE results for the pair adolescents + young adults (baseline data). Cluster label assignments for classes: adolescents + young adults using the baseline data. Center: Visualization by t-SNE. Each data point (i.e. colored circle) corresponds to a relevance map for one otolith and is overlaid with the predicted age. Darker colors on predicted ages refer to test samples. Outer images: Examples of otolith images overlaid by relevance maps for different ages predicted by the neural network.
(TIF)

S4 Fig t-SNE results for the pair adolescents + young adults (standardized data). Cluster label assignments for classes: adolescents + young adults using the standardized data. Center: Visualization by t-SNE. Each data point (i.e. colored circle) corresponds to a relevance map for one otolith and is overlaid with the predicted age. Darker colors on predicted ages refer to test samples. Outer images: Examples of otolith images overlaid by relevance maps for different ages predicted by the neural network.
(TIF)

S5 Fig t-SNE results for the pair young adults + adults (baseline data). Cluster label assignments for classes: young adults + adults using the baseline data. Center: Visualization by t-SNE. Each data point (i.e. colored circle) corresponds to a relevance map for one otolith and is overlaid with the predicted age. Darker colors on predicted age refer to test samples. Outer images: Examples of otolith images overlaid by relevance maps for different ages predicted by the neural network.
(TIF)

S6 Fig t-SNE results for the pair young adults + adults (standardized data). Cluster label assignments for classes: young adults + adults using the standardized data. Center: Visualization by t-SNE. Each data point (i.e. colored circle) corresponds to a relevance map for one otolith and is overlaid with the predicted age. Darker colors on predicted age refer to test samples. Outer images: Examples of otolith images overlaid by relevance maps for different ages predicted by the neural network.
(TIF)

## References

[1] Campana SE, Neilson J. Microstructure of fish otoliths. Canadian Journal of Fisheries and Aquatic Sciences. 1985; 42: 1014–1032.

[2] Morison AK, Burnett J, McCurdy WJ, Moksness E. Quality issues in the use of otoliths for fish age estimation. Marine and Freshwater Research. 2005; 56: 773–782.

[3] Campana SE, Thorrold SR. Otoliths, increments, and elements: keys to a comprehensive understanding of fish populations? Canadian Journal of Fisheries and Aquatic Sciences. 2001; 58(1): 30–38.

[4] Moen E, Handegard NO, Allken V, Albert OT, Harbitz A, Malde K. Automatic interpretation of otoliths using deep learning. PLoS ONE. 2018; 13(12): e0204713 10.1371/journal.pone.0204713 30557335 PMC6296523

[5] Lapuschkin S, Wäldchen S, Binder A, Montavon G, Samek W, Müller K. Unmasking Clever Hans predictors and assessing what machines really learn. arXiv:1902.10178. 2019. Available from: https://arxiv.org/abs/1902.10178. 10.1038/s41467-019-08987-4 PMC6411769 30858366

[6] Zeiler MD, Fergus R. Visualizing and Understanding Convolutional Networks. arXiv:1311.2901. 2013. Available from: https://arxiv.org/abs/1311.2901.

[7] Ribeiro MT, Singh S, Guestrin C. "Why Should I Trust You?": Explaining the Predictions of Any Classifier. arXiv:1602.04938v3. 2016. Available from: https://arxiv.org/abs/1602.04938v3.

[8] Noh H, Hong S, Han B. Learning Deconvolution Network for Semantic Segmentation. arXiv:1505.04366. 2015. Available from: https://arxiv.org/abs/1505.04366.

[9] Springenberg JT, Dosovitskiy A, Brox T, Riedmiller MA. Striving for Simplicity: The All Convolutional Net. arXiv:1412.6806. 2014. Available from: https://arxiv.org/abs/1412.6806.

[10] Simonyan K, Vedaldi A, Zisserman A. Deep Inside Convolutional Networks: Visualising Image Classification Models and Saliency Maps. arXiv:1312.6034. 2013. Available from: https://arxiv.org/abs/1312.6034.

[11] Bach S, Binder A, Montavon G, Klauschen F, Müller K, Samek W. On Pixel-Wise Explanations for Non-Linear Classifier Decisions by Layer-Wise Relevance Propagation. PLoS ONE. 2015; 10: e0130140 10.1371/journal.pone.0130140 26161953 PMC4498753

[12] Samek W, Binder A, Montavon G, Lapuschkin S, Müller K. Evaluating the Visualization of What a Deep Neural Network Has Learned. IEEE Transactions on Neural Networks and Learning Systems. 2017; 28(11): 2660–2673. 10.1109/TNNLS.2016.2599820 27576267

[13] Montavon G, Samek W, Müller K. Methods for Interpreting and Understanding Deep Neural Networks. arXiv:1706.07979. 2017. Available from: https://arxiv.org/abs/1706.07979.

[14] Szegedy C, Vanhoucke V, Ioffe S, Shlens J, Wojna Z. Rethinking the Inception Architecture for Computer Vision. arXiv:1512.00567. 2015. Available from: https://arxiv.org/abs/1512.00567.

[15] Simonyan K, Zisserman A. Very Deep Convolutional Networks for Large-Scale Image Recognition. arXiv:1409.1556. 2014. Available from: https://arxiv.org/abs/1409.1556.

[16] Deng J, Dong W, Socher R, Li LJ, Li K, Fei-Fei L. ImageNet: A large-scale hierarchical image database. IEEE Conference on Computer Vision and Pattern Recognition. 2009.

[17] Treble MA, McGowan C. Report of the Greenland halibut (Reinhardtius hippoglossoides) Age Determination Workshop. NAFO Scientific Council Studies. 2008; 41: 1–90.

[18] Albert OT. Growth and formation of annual zones in whole otoliths of Greenland halibut, a slow-growing deep-water fish. Marine and Freshwater Research. 2016; 67: 937–942.

[19] Campana SE, Annand MC, Mcmillan JI. Graphical and Statistical Methods for Determining the Consistency of Age Determinations. Transactions of The American Fisheries Society. 1995; 124: 131–138.

[20] Albert OT, Kvalsund M, Vollen T, Salberg AB. Towards Accurate Age Determination of Greenland Halibut. Journal of Northwest Atlantic Fishery Science. 2009; 40: 81–95.

[21] Frigge M, Hoaglin D, Iglewicz B. Some Implementations of the Boxplot. The American Statistician. 1989; 43(1), 50–54.

[22] Alber M, Lapuschkin S, Seegerer P, Hägele M, Schütt KT, Montavon G, et al. iNNvestigate Neural Networks. arXiv:1808.04260v1. 2018. Available from https://arxiv.org/abs/1808.04260v1.

[23] Chollet F, et al. Keras 2.1.3; 2018. https://github.com/fchollet/keras.

[24] Montavon G, Lapuschkin S, Binder A, Samek W, Müller K. Explaining nonlinear classification decisions with deep Taylor decomposition. arXiv:1512.02479. 2015. Available from https://arxiv.org/abs/1512.02479.

[25] Montavon G, Binder A, Lapuschkin S, Samek W, Müller K. Layer-Wise Relevance Propagation: An Overview. Explainable AI: Interpreting, Explaining and Visualizing Deep Learning; 2019 p.193–209.

[26] Lloyd S. Least squares quantization in PCM. IEEE Transactions on Information Theory. 1982; 28:129–136.

[27] Jolliffe I. Principal component analysis. 2^nd^ ed New York: Springer Verlag New York; 2002.

[28] Van der Maaten L, Hinton G. Visualizing data using t-SNE. Journal of Machine Learning Research. 2008; 9: 2579–2605.

[29] Von Luxburg UV. A tutorial on spectral clustering. arXiv:0711.0189. 2007. Available from: https://arxiv.org/abs/0711.0189.

[30] Chinchor N. MUC-4 Evaluation Metrics. in Proc. of the Fourth Message Understanding Conference.1992.

[31] Harbitz A, Albert OT. Pitfalls in stock discrimination by shape analysis of otolith contours. ICES Journal of Marine Science. 2005; 72(7): 2090–2097.

[32] Madhun AS, Karlsen Ø, Karlsbakk E. Annual report on health monitoring of wild anadromous salmonids in Norway 2018-screening of migrating Atlantic salmon (Salmo salar) postsmolts from the Trondheim fjord for viral infections. Rapport from the Institute of Marine Research 2019 Available from https://www.hi.no/en/hi/nettrapporter/rapport-fra-havforskningen-en-2019-28.

[33] Frie AK, Fagerheim KA, Hammill MO, Kapel FO, Lockyer C, Stenson GB, et al Error patterns in age estimation of harp seals (Pagophilus groenlandicus): results from a transatlantic, image-based, blind-reading experiment using known-age teeth. ICES Journal of Marine Science. 2011; 68(9): 1942–1953.

[34] Stransky C, Baumann H, Fevolden SE, Harbitz A, Høie H, Nedreaas KH, et al Separation of Norwegian coastal cod and Northeast Arctic cod by outer otolith shape analysis. Fisheries Research. 2008; 90: 26–35.

