# Supplementary figures and images for: Explaining decisions of deep neural networks used for fish age prediction
Source: PLoS One. 2020 Jun 19;15(6):e0235013. doi: 10.1371/journal.pone.0235013 (PMC7304622; doi:10.1371/journal.pone.0235013)

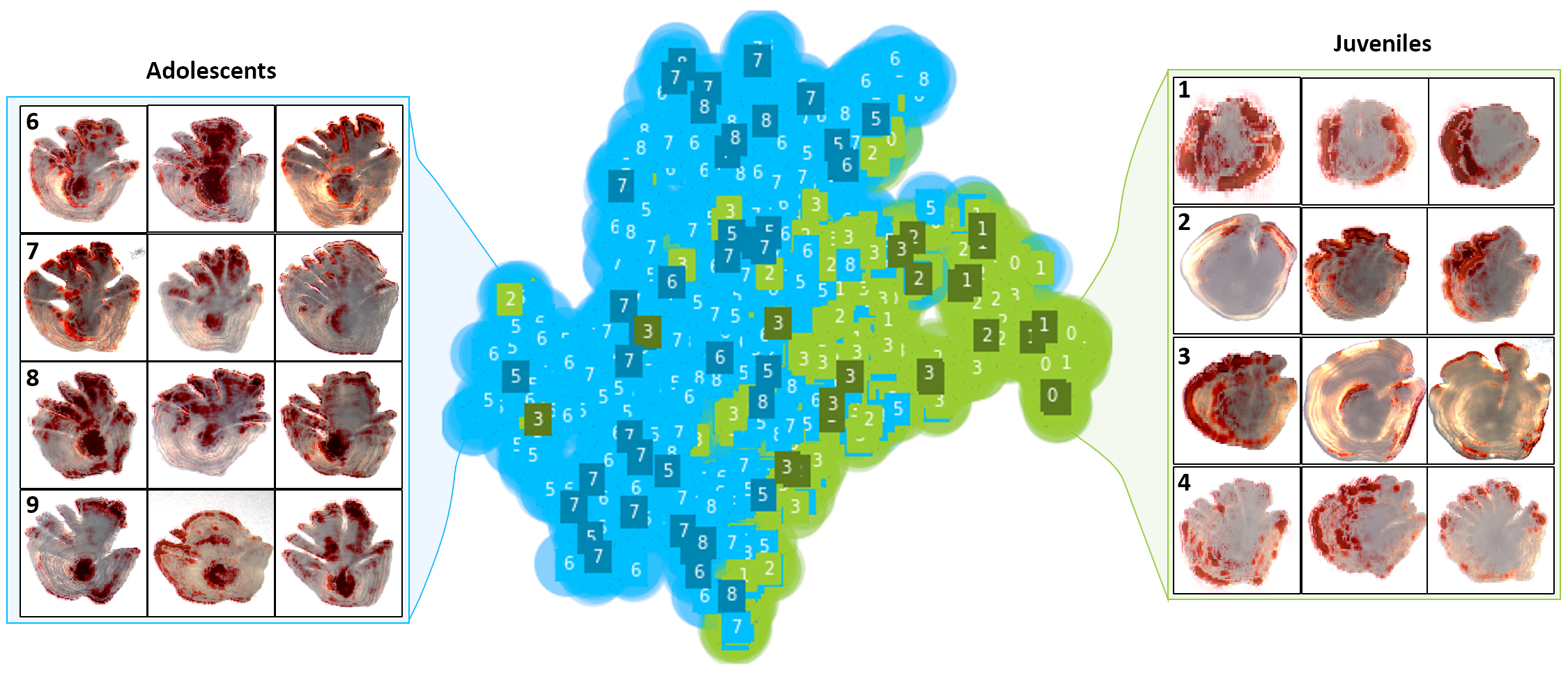

Supplement: S1 Fig — Cluster label assignments for classes: juveniles + adolescents using the baseline data. Center: Visualization by t-SNE. Each data point (i.e. colored circle) corresponds to a relevance map for one otolith and is overlaid with the predicted age. Darker colors on predicted ages refer to test samples. Outer images: Examples of otolith images overlaid by relevance maps for different ages predicted by the neural network. (TIF) [file pone.0235013.s001.tif]

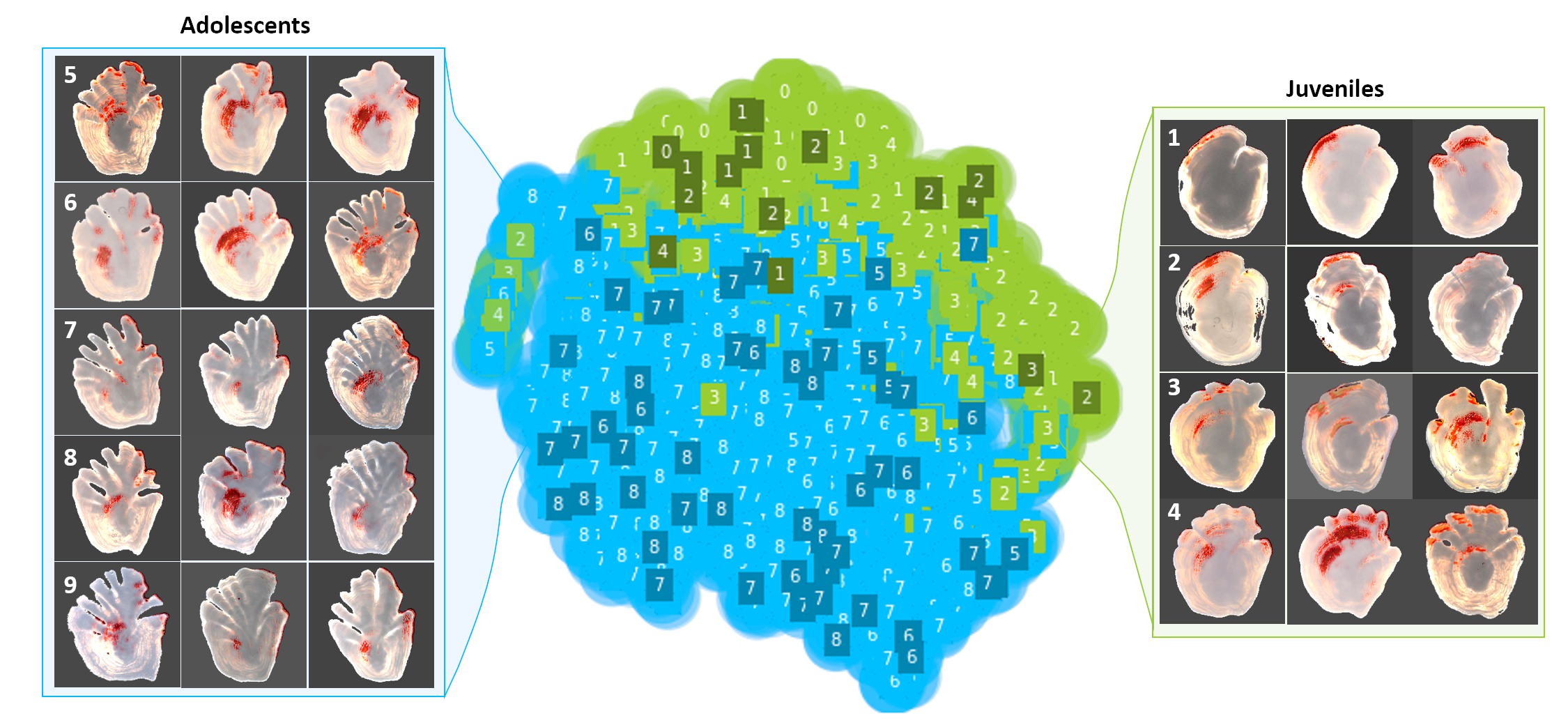

Supplement: S2 Fig — Cluster label assignments for classes: juveniles + adolescents using the standardized data. Center: Visualization by t-SNE. Each data point (i.e. colored circle) corresponds to a relevance map for one otolith and is overlaid with the predicted age. Darker colors on predicted ages refer to test samples. Outer images: Examples of otolith images overlaid by relevance maps for different ages predicted by the neural network. (TIF) [file pone.0235013.s002.tif]

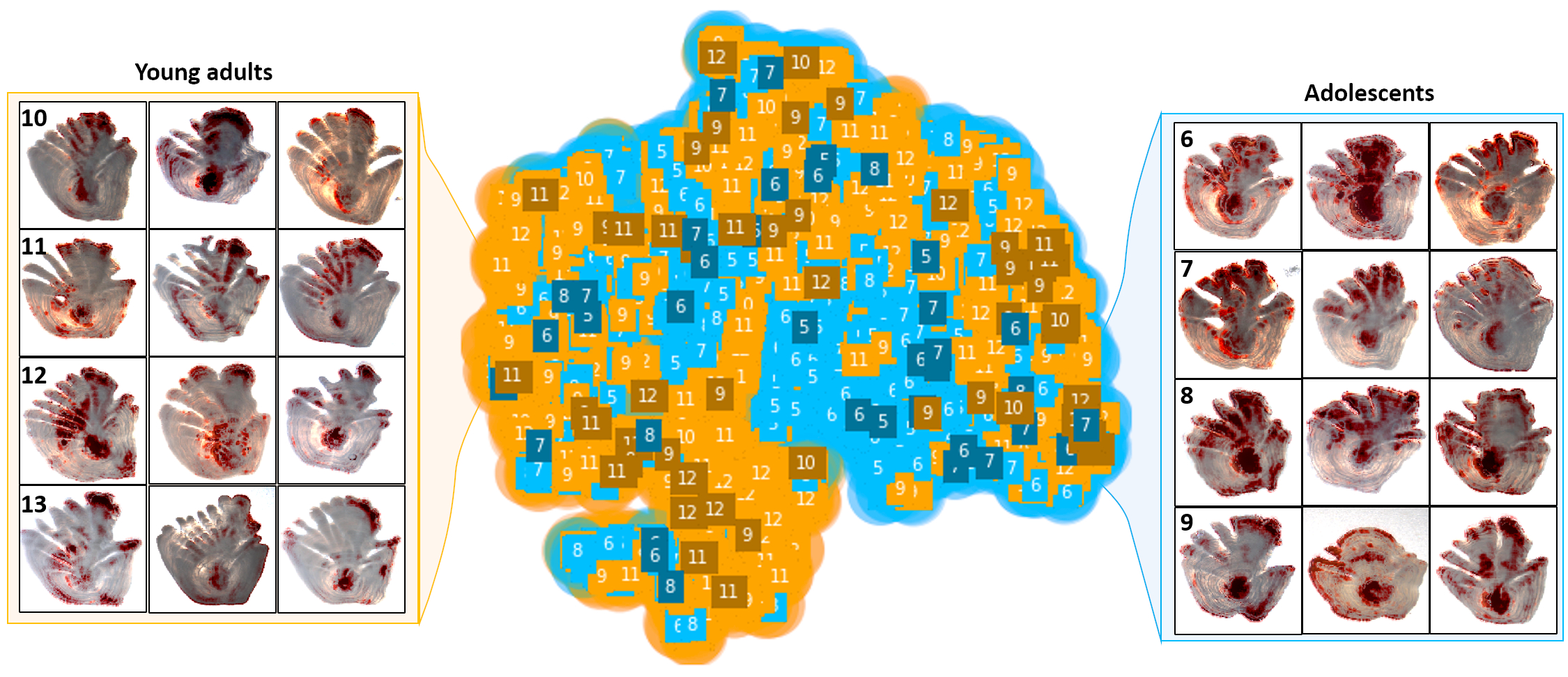

Supplement: S3 Fig — Cluster label assignments for classes: adolescents + young adults using the baseline data. Center: Visualization by t-SNE. Each data point (i.e. colored circle) corresponds to a relevance map for one otolith and is overlaid with the predicted age. Darker colors on predicted ages refer to test samples. Outer images: Examples of otolith images overlaid by relevance maps for different ages predicted by the neural network. (TIF) [file pone.0235013.s003.tif]

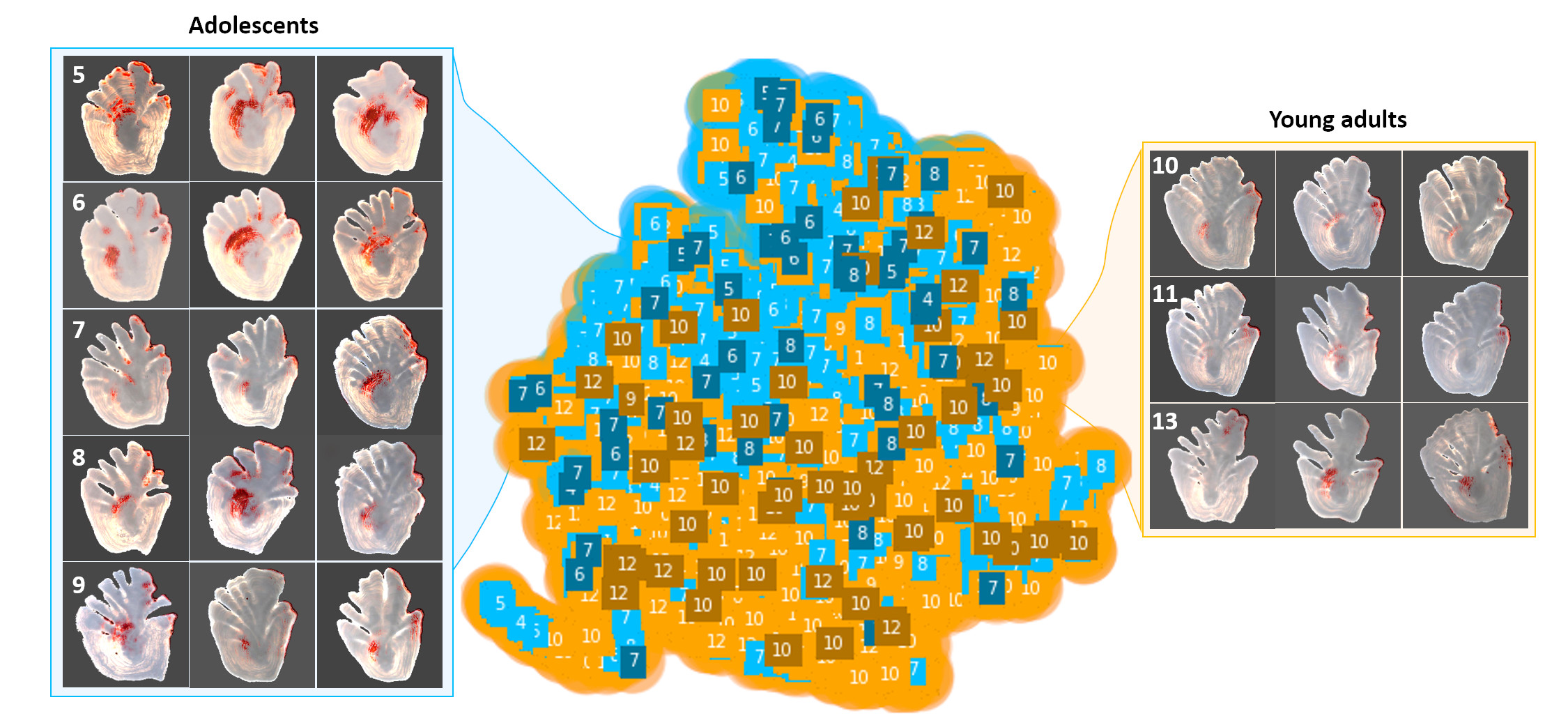

Supplement: S4 Fig — Cluster label assignments for classes: adolescents + young adults using the standardized data. Center: Visualization by t-SNE. Each data point (i.e. colored circle) corresponds to a relevance map for one otolith and is overlaid with the predicted age. Darker colors on predicted ages refer to test samples. Outer images: Examples of otolith images overlaid by relevance maps for different ages predicted by the neural network. (TIF) [file pone.0235013.s004.tif]

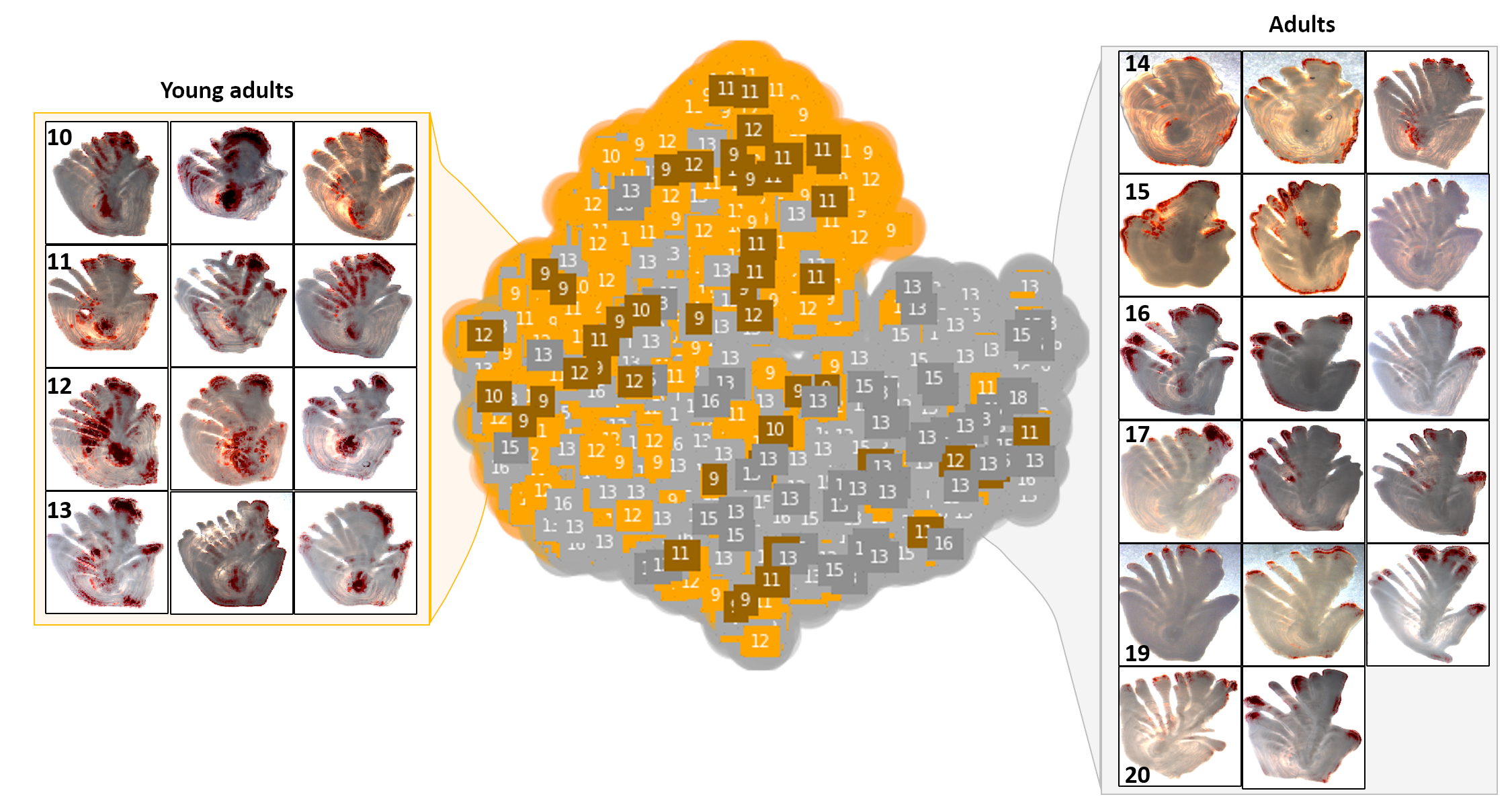

Supplement: S5 Fig — Cluster label assignments for classes: young adults + adults using the baseline data. Center: Visualization by t-SNE. Each data point (i.e. colored circle) corresponds to a relevance map for one otolith and is overlaid with the predicted age. Darker colors on predicted age refer to test samples. Outer images: Examples of otolith images overlaid by relevance maps for different ages predicted by the neural network. (TIF) [file pone.0235013.s005.tif]

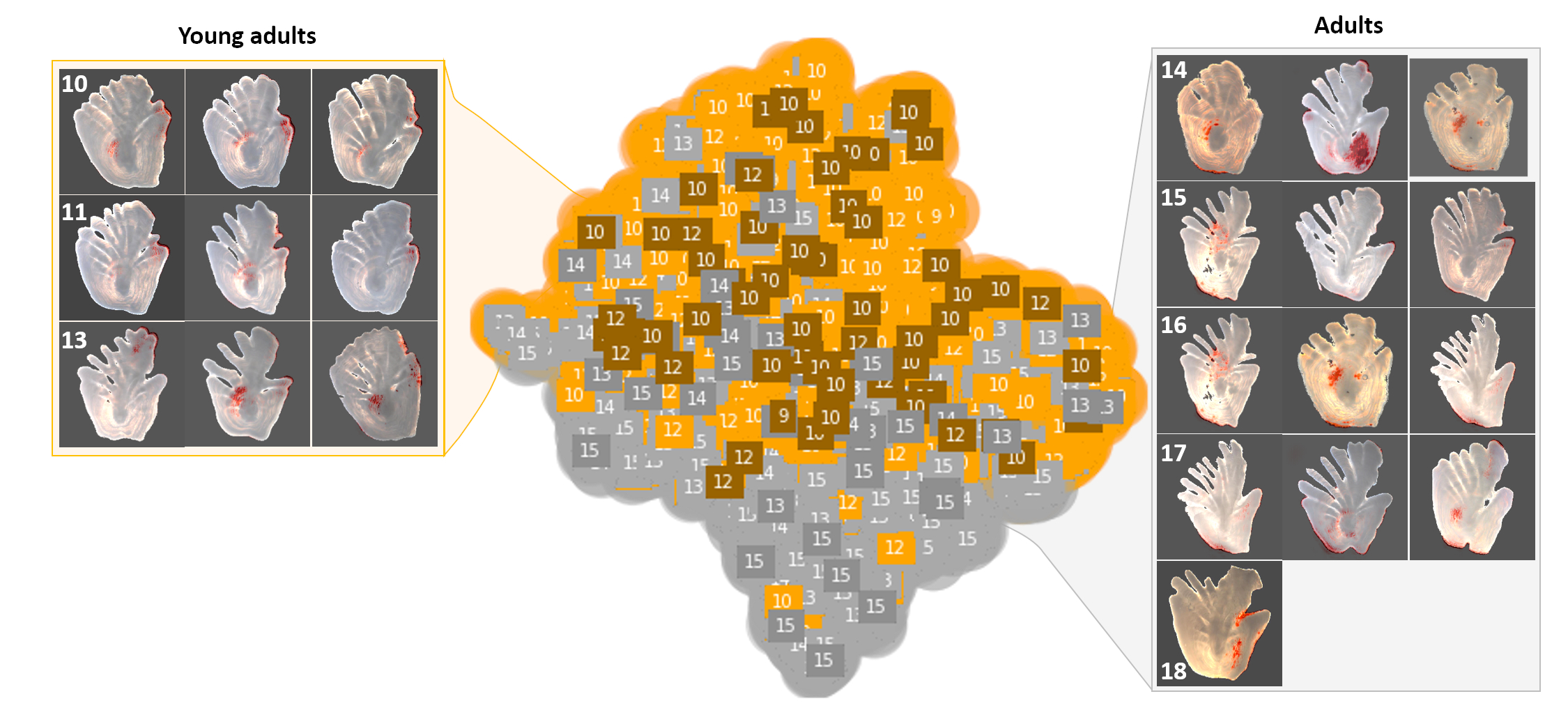

Supplement: S6 Fig — Cluster label assignments for classes: young adults + adults using the standardized data. Center: Visualization by t-SNE. Each data point (i.e. colored circle) corresponds to a relevance map for one otolith and is overlaid with the predicted age. Darker colors on predicted age refer to test samples. Outer images: Examples of otolith images overlaid by relevance maps for different ages predicted by the neural network. (TIF) [file pone.0235013.s006.tif]
